# Therapeutic Potential of Antioxidants and Hybrid TEMPOL Derivatives in Ocular Neurodegenerative Diseases: A Glimpse into the Future

**DOI:** 10.3390/biomedicines11112959

**Published:** 2023-11-01

**Authors:** Charles E. Amankwa, Bindu Kodati, Nina Donkor, Suchismita Acharya

**Affiliations:** 1 Department of Pharmacology and Neuroscience, University of North Texas Health Science Center, Fort Worth, TX 76107, USA; charlesamankwa@my.unthsc.edu (C.E.A.); bindu.kodati@unthsc.edu (B.K.); ninadonkor@my.unthsc.edu (N.D.); 2 North Texas Eye Research Institute, University of North Texas Health Science Center, Fort Worth, TX 76107, USA; 3 Department of Pharmaceutical Science, University of North Texas Health Science Center, Fort Worth, TX 76107, USA

**Keywords:** nitric oxide, reactive oxygen species, glaucoma, oxidative stress, TEMPOL derivatives, neuroprotection, trabecular meshwork, retina ganglion cells, hybrid small molecules, SA-2, SA-9, SA-10

## Abstract

Reactive oxygen species play a significant role in the pathogenesis of various ocular neurodegenerative diseases especially glaucoma, age-related macular degeneration (AMD), and ocular ischemic stroke. Increased oxidative stress and the accumulation of ROS have been implicated in the progression of these diseases. As a result, there has been growing interest in exploring potential therapeutic and prophylactic strategies involving exogenous antioxidants. In recent years, there have been significant advancements in the development of synthetic therapeutic antioxidants for targeting reactive oxygen species (ROS) in neurodegenerative diseases. One area of focus has been the development of hybrid TEMPOL derivatives. In the context of ocular diseases, the application of next-generation hybrid TEMPOL antioxidants may offer new avenues for neuroprotection. By targeting ROS and reducing oxidative stress in the retina and optic nerve, these compounds have the potential to preserve retinal ganglion cells and trabecular meshwork and protect against optic nerve damage, mitigating irreversible blindness associated with these diseases. This review seeks to highlight the potential impact of hybrid TEMPOL antioxidants and their derivatives on ocular neurodegenerative disorders.

## 1. Introduction

The retina is a functional unit of the central nervous system (CNS) constituted by populations of neurons that are connected via axons of the optic nerve to the brain. These neurons and complex synaptic connections that underlie the visual system have long been known to be susceptible to the negative effects of reactive oxygen species (ROS) [1,2,3,4]. This is due to inherent features such as high metabolic rate, high intracellular concentrations of transition metals that can catalyze the formation of reactive hydroxyl radicals, low antioxidant levels, and the high composition of retinal fatty acids that are susceptible to peroxidation [4]. These cumulatively results in damage to the retina and potentially impacts the development of neurodegenerative diseases (NDs). Neurodegenerative diseases (NDs) comprise the complex and slow progressive loss of neuronal cells resulting in nervous system dysfunction. More specifically, ocular neurodegenerative diseases refer to a group of disorders characterized by the progressive degeneration of neuronal cells in the eye, leading to vision impairment or loss. These diseases include age-related macular degeneration (AMD), glaucoma, and retinitis pigmentosa, among others [1,2]. Despite their distinct clinical manifestations, these conditions share common pathophysiological mechanisms, including oxidative stress, inflammation, and impaired cellular homeostasis. Over the years, extensive research has focused on understanding the underlying causes of ocular neurodegenerative diseases and developing effective treatment strategies. Cumulative studies have highlighted the role of genetics, protein misfolding, impaired energy metabolism, imbalanced redox homeostasis, and neuroinflammation as key in the pathophysiological triggers of these NDs. Despite their apparently diverse mechanisms, these pathophysiologic mechanisms appear to converge into oxidative stress as a key regulator.

Oxidative stress (OS) is one of the commonly reported pathways involved in the pathogenesis of ocular neurodegenerative diseases. Particularly in the context of glaucoma, putative biomarkers such as interleukin-6 (IL-6) and malonyl dialdehyde (MDA) have been identified in the aqueous humor (AH) of patients with primary open angle glaucoma (POAG), indicative of oxidative stress-induced lipid peroxidation and inflammation [4,5,6].

The unbalanced release of reactive oxygen/nitrogen species (RONS) and/or the inability of biological systems to detoxify reactive products produce oxidative damage to target tissues. These ROS represent a variety of molecules and free radicals derived from molecular oxygen. At the ground state, molecular oxygen is a bi-radical with two single electrons in its outer shell. During cellular activities, the reduction in molecular oxygen in water leads to the formation of fairly stable intermediate products and subsequently the generation of superoxide anion (O_2_^·−^): a precursor of most ROS. Other examples of ROS include hydrogen peroxide (H_2_O_2_), hydroxyl radical (•OH), and singlet oxygen (^1^O_2_). These agents can potentially react with either endogenously or exogenously generated nitric oxide (NO) to produce the readily toxic peroxynitrite (ONOO), a potent mediator of OS [7].

Collectively, RONS are generated in biological systems as metabolic by-products derived from cellular functions in the mitochondria, peroxisomes, endoplasmic reticulum, and phagocytic cells. Excessive production of RONS can cause oxidative damage to biomolecules such as lipids, nucleic acids, and proteins, which may alter cellular structure and function. Furthermore, environmental factors such as ultraviolet radiations, pollutants, and heavy metals as well as exogenous xenobiotics such as antiblastic drugs have been reported to contribute to increased RONS production. In mammalians, enzymes such as NADPH-oxidase, myeloperoxidase, and nitric oxide synthase (NOS) are known to further promote the generation of RONS. The controlled production of generated RONS in the extracellular space by these enzymes elicits beneficial effects as they form part of the innate immune system to kill invading bacteria. However, an overwhelming release of RONS induces deleterious effects such as apoptosis, lipid peroxidation, protein oxidation, and damage to deoxyribonucleic acid (DNA).

With an aging global population, neurological disorders are predicted to cause enormous economic and health challenges given their current contribution of 6.3% of the global burden of all diseases [8]. POAG is an example of such an ocular neurodegenerative disorder with an estimated global prevalence of approximately 112 million cases by the year 2040 with at least 6 to 8 million patients over 40 years becoming bilaterally blind [3,9]. While NDs appear to develop late, juvenile forms occur after birth and may damage the central nervous system (CNS), potentially affecting memory, reasoning, motor skills, and vision [10,11].

**Figure 1:**
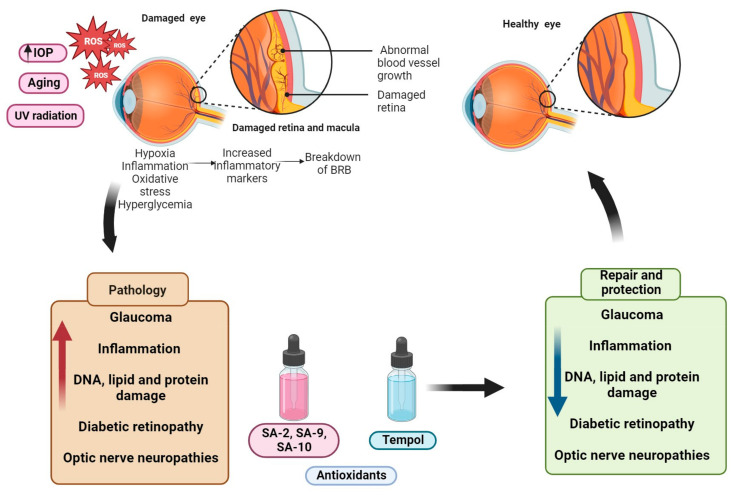
Graphical representation of antioxidants as possible therapeutic alternatives for ocular neurodegenerative diseases. Excessive ROS generated from aging, UV radiation, and elevated IOP among others results in the development of oxidative stress in ocular tissues. The release of these free radicals activates inflammation, hypoxia, and damages the retina. Ultimately, pathological processes such as inflammation and optic neuropathies are triggered. To circumvent or slow down these cascade of events, superoxide dismutase (SOD) mimetic small molecule radical scavenger TEMPOL or its hybrid TEMPOL antioxidant therapies are reported to demonstrate promising therapeutic benefits in oxidative stress-mediated ocular diseases.

Current therapeutic options for neurogenerative diseases are symptomatic with modest efficacies. Thus, exploring robust alternatives that target oxidative stress pathways is essential to providing lasting solutions for ocular NDs and perhaps NDs at large (Figure 1) [12]. Herein, we review the impact of oxidative stress on common signaling pathways of some ocular NDs with specific focus on glaucoma. In addition, we review both existing antioxidants and emerging TEMPOL-derived small molecules with promising pre-clinical effects in the management of some ocular NDs.

## 2. Mechanisms and Signaling Pathways Involved in Ocular Neurodegenerative Diseases

The progressive loss of retinal ganglion cells (RGCs) and photoreceptors is attributed to several contributory mechanisms. Some elucidated pathways include the oxidative stress pathway, mitochondrial dysfunction, excitotoxicity, protein misfolding, Wnt/β catenin signaling, and phosphatidylinositide-3-kinases (PI3K)/protein kinase B (AKT)/the mammalian target of rapamycin (mTOR) pathways. These combined with the failure to detoxify systems converge to initiate a cascade of events that culminates in cell death, ultimately leading to retinal neurodegeneration and vision impairment [13,14,15,16]. In this review, we explore some common oxidative stress-mediated pathways in age-related macular degeneration, diabetic retinopathy, and glaucoma.

### 2.1. Oxidative Stress Pathways in Ocular Neurodegenerative Diseases

**Figure 2:**
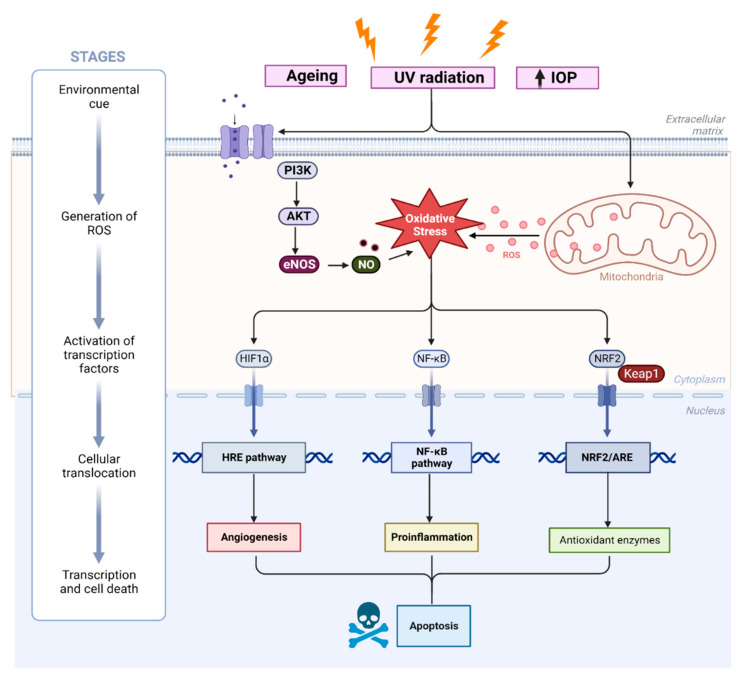
A schematic diagram depicting a summary of some signaling pathways in oxidative stress. Apart from intracellular signals, such as cellular energy status and hypoxia stress, extracellular cues including aging, high IOP, and UV radiation play essential roles in activating ROS generation intracellularly. PI3/AKT/mTOR pathways are activated, which activates downstream transcription factors such as the hypoxia response element (HRE), NF-KB, and NRF2/ARE pathways. These pathways induce angiogenesis, inflammation, and potentially inhibit antioxidant enzyme systems which culminate in apoptosis.

Reactive oxygen species (ROS)-mediated neurodegeneration is triggered by the abnormal modulation in the functions of biomolecules and consequently the activation of many signaling pathways. The redox biology of oxidative stress involves elevation of RONS and/or the concurrent attenuation of antioxidant enzyme systems. This phenomenon targets different substrates (e.g., lipids, DNA etc.) and signaling pathways for cellular survival, thus resulting in the acceleration of apoptotic and non-apoptotic cell death [17]. Interestingly, ocular tissues involving the trabecular meshwork (TM), optic nerve head (ONH), and retina are susceptible to RONS-induced cell damage. These RONS result in the activation of the nuclear factor E2-related factor 2 (Nrf2)/antioxidant response element (ARE) pathway, which regulates the expression of antioxidant enzymes. RONS and electrophiles react with cysteine residues present on repressor protein Kelch-like ECH-associated protein-1 (keap 1), inducing a conformational change that liberates Nrf2 and inhibits ubiquitination, hence allowing Nrf2 to translocate into the nucleus to activate phase II genes (Figure 2). In addition, PI3K/AKT and nuclear factor-κB (NF-κB) signaling are also activated to control vascular tone through the production of NO as well as regulate cellular proliferation and apoptosis in inflammatory states, respectively. The mitogen-activated protein kinases (MAPK) family plays a notable role in ROS generation. This pathway includes p38, MAPK, extracellular regulated kinase (ERK1/2), and c-Jun N-terminal kinase (JNK) (Figure 2). ERK1/2 regulates the expression of the NOX4 gene and the activation of JNK, and its substrate c-Jun, further induces the expression of ROS that acts as an important secondary messenger and modulator for the ERK1/2/NOX4/ROS and JNK/c-Jun pathway [18].

### 2.2. Oxidative Stress in Glaucoma

While increased IOP is a fundamental factor in the development of glaucoma, experimental evidence suggests a significant contribution of oxidative stress in accelerating vision loss and apoptosis [14]. In the anterior chamber, the trabecular meshwork (TM) is among the most susceptible tissues to oxidative damage partly due to its relatively lower levels of antioxidant defense systems compared to both the cornea and iris [19,20]. In POAG, one of the profound mechanisms of TM and RGC death is mediated through mitochondrial dysfunction often as a consequence of factors such as elevated mechanical stress and hypoxia. Mitochondrial dysfunction may lead to the activation of NADPH oxidase, an ROS-producing enzyme that in turn triggers apoptosis downstream [21,22,23]. In both in vivo and in vitro studies, the vasoactive peptide: endothelin 1 (ET-1) treatment showed a decline in the ATP5H and cytochrome c oxidase (COX17) in primary rat RGCs. The decreased levels of the mitochondrial proteins ATP5H and COX17 are significantly reflected in a decline in oxidative phosphorylation, leading to a potential increase in OS [24].

Moreover, the only modifiable risk factor, elevated IOP, has been shown to result in oxidative stress, which triggers the activation of ATP-sensitive potassium (KATP) channels in retinal vessels leading to vasotoxicity by the increased stimulation of p2X7 receptor. This culminates in an increase in intracellular calcium levels [25,26]. In addition, McElnea et al. reported elevated ROS production, impaired mitochondrial function, and elevated cytosolic Ca^2+^ [27]. These glaucomatous paradigms either decrease OXPHOS and/or glycolysis in RGCs and TM impairing their morphology, viability, and functionality [22,24]. Glaucomatous damage has also been reported to elevate the nitric oxide (NO) production from glial cells (pathologic concentrations), which interacts with superoxide to form peroxynitrite that mediates the peroxidation and nitrotyrosylation of biomolecules. In addition, neuroinflammation can be induced from increased ROS levels by stimulating interleukins and tumor necrosis factor (TNF-α). In a co-culture of rat RGCs and glial cells, Tezel et al. studied the influences of glial cells on the survival of RGCs after exposure to different stress conditions with simulated ischemia and elevated hydrostatic pressure. Unsurprisingly, TNF-α-induced apoptosis was seen in RGCs after incubation of the cocultures in the presence of simulated ischemia or elevated hydrostatic pressure for 72 h. Morphological changes including cell body shrinkage and compaction of the nucleus were also observed [28].

### 2.3. Oxidative Stress in Age-Related Macular Degeneration

Age-related macular degeneration (AMD) is a complex and progressive degenerative disorder of the macular, a region of the central retina responsible for the highest visual acuity. The aberrant accumulation of lipids and proteins between the Bruch’s membrane and retinal pigmented epithelium (RPE), loss of photoreceptor cells, and neovascularization are among the main pathological features of AMD. However, the exact underlying mechanism for the development of AMD remains unknown. A cascade of sequential events from several factors, including metabolic changes, inflammation, genetic predisposition, and oxidative stress, are thought to play a critical role in the pathophysiology of AMD [29]. There is a documented increase in lipofuscin, MDA, and 8-hydroxy deoxy guanosine (8-OHdG) in the blood serum of AMD subjects. In addition, histopathological studies have demonstrated an association between high levels of lipofuscin and the degeneration of RPE cells and the adjacent photoreceptors. Carboxyethyl pyrrole, a lipid peroxidation product formed from docosahexaenoic acid (DHA) in Bruch’s membrane, has also been noticed in the ageing retina, indicative of oxidative stress [29,30,31].

### 2.4. Oxidative Stress in Diabetic Retinopathy

Diabetes Mellitus (DM) is a metabolic disease characterized by hyperglycemia due to insufficient secretion of insulin (type 1 DM), insulin resistance (type 2 DM) or both. Microvascular complications of untreated or poorly managed DM may trigger retinal abnormalities including neovascularization, macular edema, and diabetes retinopathy (DR). While DM plays a critical role in the progression of DR, the exact mechanism remains unclear [32]. Compelling evidence from both experimental and clinical studies suggest the close interplay of hyperglycemia and oxidative stress is key etiological pathway in the progression of DR. ROS generated secondary to hyperglycemia tend to damage biomolecules such as DNA, proteins, and lipid membranes. Notably, the retina and the TM are highly susceptible to damage by oxidative stress [22,33,34,35,36].

In diabetic retinopathy, the exposure of photosensitizer molecules such as porphyrins and phthalocyanines to radiant energy may create an environment conducive for the generation of ROS. With elevated metabolic activities and high oxygen consumption rates in DR, the vascular endothelium is more susceptible to lipid peroxidation, where the polyunsaturated fatty acids such as docosahexaenoic acid and arachidonic acid are damaged, releasing toxic biomarkers (such as MDA, acrolein) [37,38,39,40].

## 3. Some Recent Advancements in Glaucoma Therapy

Amongst the several risk factors of glaucoma, elevated IOP is the only known modifiable risk factor. Increased IOP leads to a buildup of abnormal pressure in the anterior chamber, which results in ischemia, decreased oxygen supply, and progressive retinopathy. Over the past 100 years, the basic treatment and surgical paradigms for glaucoma has focused on slowing down the progression of the disease by reducing aqueous humor (AH) production and/or promoting its outflow primarily through the uveoscleral outflow [41,42]. In principle, AH is formed by the ciliary processes as a result of active transport of solutes over the double-layered non-pigmented ciliary epithelium. The AH produced is drained out from the eye via two different pathways at the iridocorneal angle, namely, uveoscleral and conventional pathways. For the conventional pathway, AH diffuses into the Schlemm’s canal by way of the trabecular meshwork and then drains via the collector channels into the episcleral veins Interestingly, the conventional pathway contributes to more than 80% AH outflow while the uveoscleral pathway, on the other hand, contributes to nearly 15–20% AH drainage. In the uveoscleral pathway, AH passes through muscle bundles into the supraciliary and suprachoroidal spaces, from which it is drained through the sclera. Topical prostaglandins (PGs) have been the gold standard therapeutic agent employed in the treatment armamentarium due to their powerful ocular hypotensive effect and overall good tolerability profile [41]. However, the majority of the IOP-lowering medications mainly target the uveoscleral pathway with modest efficacies. Below are some commonly utilized IOP-lowering medications for managing glaucoma.

### 3.1. Increasing AH outflow and Decreasing AH Formation

**Figure 3:**
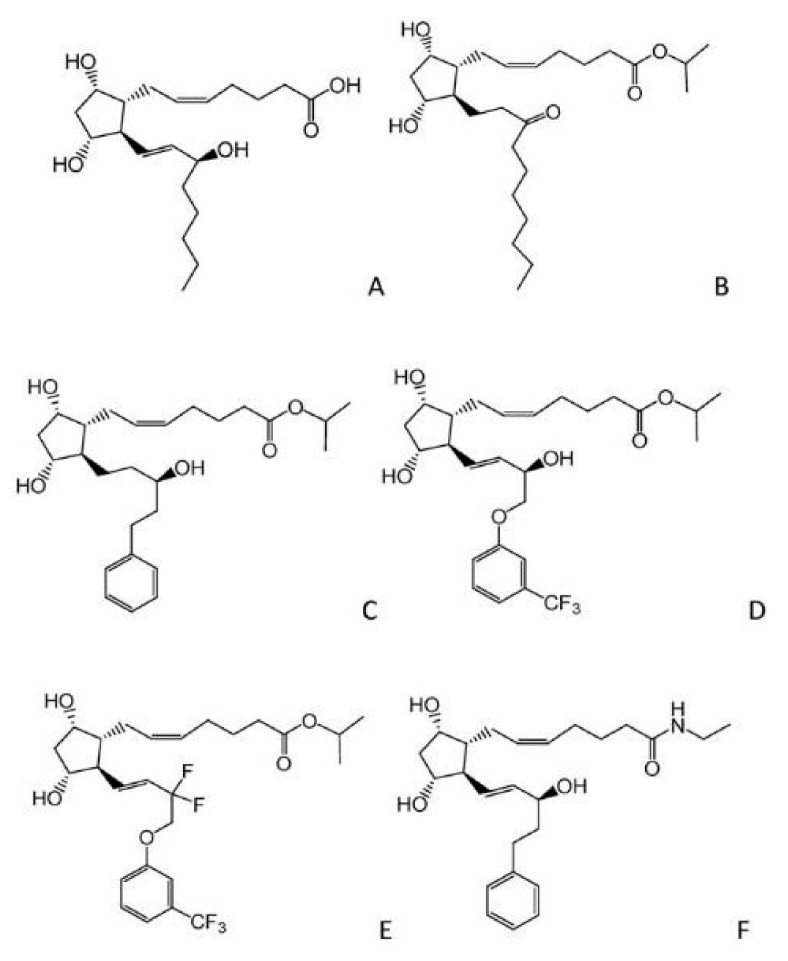
Molecular structure of synthetic prostaglandin (PG) analogues (**A**) PGF2α, (**B**) unoprostone, (**C**) latanoprost, (**D**) travoprost, (**E**) tafluprost, (**F**) bimatoprost.

Latanoprost is an ester prodrug of prostaglandin PGF2α and the first marketed prostaglandin analog for reducing IOP in patients with open-angle glaucoma or ocular hypertension [42,43,44]. Subsequently, bimatoprost (Lumigan^®^, 0.03%, Irvine, CA, USA), travoprost (Travatan^®^, 0.004%, Fort Worth, TX, USA), tafluprost (Zioptan^®^, 0.0015%, Rahway, NJ, USA), and the partial agonist, unoprostone (Rescula^®^, 0.15%, Basel, Switzerland) were also approved (Figure 3). These prostaglandin analogues exert a robust IOP-lowering effect (20–35%) in pre-clinical animal models. Their mode of action primarily involves the stimulation of AH outflow through the interstitial spaces of the ciliary muscle into the suprachoroidal space. This pathway (uveoscleral pathway) allows for about 10–20% of the total AH drainage. In addition, growing bodies of evidence have reported the role of latanoprost acid in the uveoscleral pathway by activating the expression of matrix metalloproteinases (MMP 1, 3, and 9) in the ciliary processes [45]. This mechanism leads to the remodeling of the extracellular matrix and subsequently increasing AH outflow facility through the non-conventional pathway. β-blockers including timolol and betaxolol decrease IOP by lowering AH formation [46].

### 3.2. Alpha-2 Receptor Agonists

Alpha-2 adrenergic receptor agonists have been used as IOP-lowering drugs primarily via the uveoscleral pathway. Mechanistically, α_2_ adrenergic agonists reduce AH production by inhibiting adenylate-cyclase, which leads to decreased cAMP levels. Examples of drugs in this category include clonidine, apraclonidine, and brimonidine. Despite the marked IOP reducing effect of α_2_ adrenergic agonists, studies have shown promising neuroprotective properties of brimonidine in a variety of ocular models [47,48,49]. Interestingly, the three isoforms of α_2_ agonists are expressed in ocular tissues, suggesting their contribution to IOP homeostasis and perhaps neuroprotection. α_2A_-adrenergic receptors have been identified in the non-pigmented ciliary epithelium while α_2B_ and α_2c_ are expressed in axons and photoreceptors, respectively [47]. In addition, Gao et al. showed in a randomized pre-clinical study that intravitreally injected bimonidine in Sprague–Dawley rats demonstrated increased levels of brain-derived neurotrophic factors (BDNF)-positive RGC counts (55–166%) when compared to control [50]. Brimonidine also provided neuroprotection in rat models of ischemia-induced RGC death and an optic nerve crush model of mechanical injury. It is believed that its neuroprotective effect is mediated by alpha-2 adrenergic receptor activation, leading to inhibition of pro-apoptotic signaling and the activation of anti-apoptotic genes [51]. However, the true mechanism by which stimulation of α_2_ receptors elicit brimonidine’s neuroprotective response in RGCs remains unclear.

### 3.3. Nitric Oxide Donors

While prostaglandin analogs offer great IOP-lowering activity in POAG patients, there is no reported activity on the conventional/trabecular meshwork outflow pathway, which contributes to approximately 80–90% AH drainage. In addition, a proportion of patients expressing normotension glaucoma do not respond to latanoprost monotherapy, which leaves a grey area in the holistic management of all subtypes of glaucoma [52,53]. Growing bodies of evidence have shown that nitric oxide (NO) plays a significant role in the regulation of IOP homeostasis by directly/indirectly increasing AH outflow via the trabecular meshwork, thereby lowering IOP through the conventional outflow pathway. The trabecular meshwork is a porous fenestrated tissue of approximately 350 × 50–150 µm in cross-section boarded by the Schlemm’s canal. It is composed of three regions that define the filtering portion of the TM namely: the uveal meshwork, corneoscleral meshwork, and the juxtacanalicular region (JCT). The cells of the outer layer of the TM primarily act as pre-filters that aggressively phagocytize cellular debris from AH before the fluid percolates deeper into the site of highest AH resistance (JCT). Invariably, agents that improve drainage through the TM possess the potential of lowering IOP significantly.

**Figure 4:**
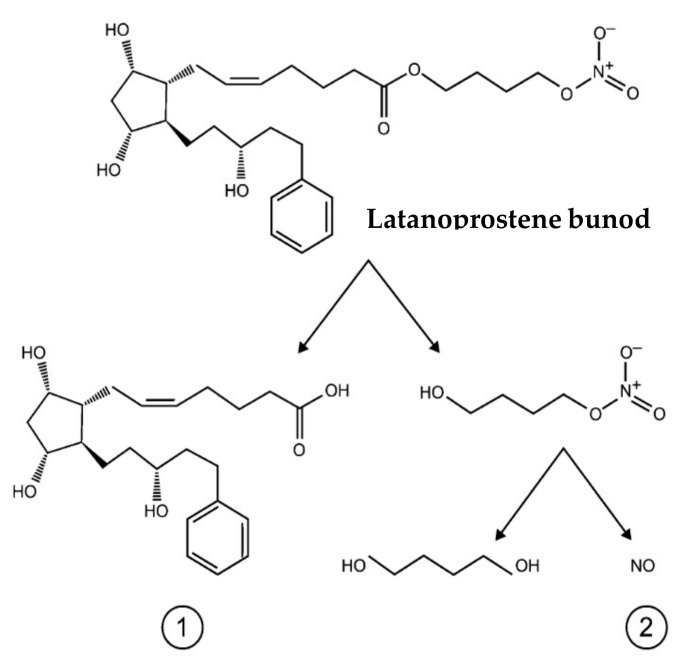
Chemical structure of latanoprostene bunod (Vyzulta) depicting the hydrolysis of the ester prodrug to two active products (**1**) latanoprost acid (**2**) nitric oxide donor that subsequently release NO and the inactive metabolite 1,4-butanediol.

**Figure 5:**
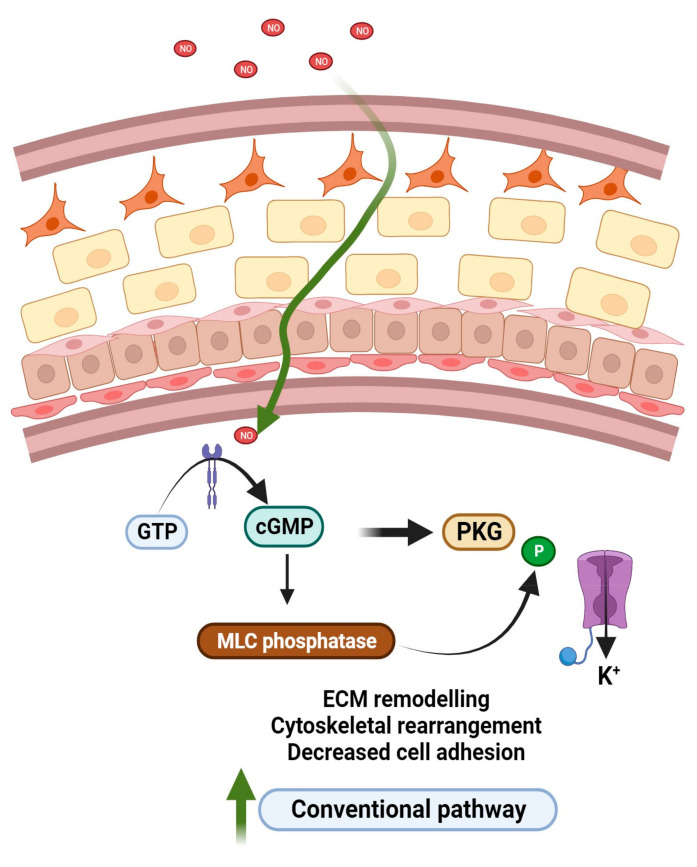
Schematic diagram showing the mechanism of action of NO donating compounds in the trabecular meshwork. NO is a 30 Da diatomic gasotransmitter that easily penetrates the TM and activates the heterodimeric enzyme soluble guanylate cyclase, and results in the downstream activation of cyclic guanosine monophosphate (cGMP). This results in the phosphorylation of protein kinase G thus allowing the efflux of potassium that causes relaxation of the TM thereby promoting outflow through the conventional pathway.

Taking advantage of this mechanism, newly designed NO-donating drugs combine the IOP-lowering effect of latanoprost with the increased trabecular meshwork AH outflow effect of NO [43,44,54,55,56]. NO is an endogenous diatomic, lipophilic signaling molecule with a light weight (MW = 30 D). NO is produced by a family of nitric oxide synthases (NOS) made of three isozymes, namely neuronal NOS (nNOS, NOS1), inducible NOS (iNOS, NOS2), and endothelial NOS (eNOS, NOS3) [56,57]. Under physiologic conditions, the constitutively expressed isoforms NOS 1 and NOS 3 mediate the conversion of L-arginine to L-citrulline and NO, while NOS 2 is produced under pathological conditions in response to immune activation. NO activates the heterodimeric enzyme guanylyl cyclase resulting in the downstream activation of the second messenger cyclic guanosine monophosphate (cGMP) resulting in the activation of protein kinase G (PKG), which further activates the ubiquitous big K+ channels allowing for the efflux of K+ to cause a relaxation of the trabecular meshwork [54,55,57]. In addition, Shahidullah et al. in an experimental study proposed an alternate mechanism of action for sodium nitroprusside (SNP) in porcine eyes. They showed that NO reduced AH by activation of the Src family kinases in NaK-ATPase transporters in the ciliary processes [58]. Recently, latanoprostene bunod (Vyzulta TM Bausch and Lomb, Rochester, NY, USA) was approved by the United State Food and Drug Administration (USFDA) in 2017 for the management of POAG and ocular hypertension. Latanoprostene bunod (LBN) lowers IOP via a dual mechanism. LBN is metabolized into two distinct moieties upon administration: latanoprost acid and butanediol mononitrate (Figure 4). With the established role of latanoprost acid as a prostaglandin F2 alpha analog, it acts by selectively activating PGF2 (FP) receptors expressed abundantly in the ciliary muscle, sclera, and ciliary epithelium. The latanoprost acid moiety primarily increases uveoscleral outflow by increasing the expression of matrix metalloproteinases (MMPs) such as MMP I, III, and IX, which enhances degradation of collagen type I, III, and IV in the longitudinal bundles of the ciliary muscles and sclera regions. Butanediol mononitrate on the other hand is metabolized to NO and the inactive moiety 1,4-butanediol. NO released induces reduction in the cell volume and contractility of vascular smooth muscles upon activation of the sGC/cGMP/PKG cascade pathway. NO can easily penetrate the TM and inner wall of Schlemm’s canal (SC), causing decreased myosin light chain-2 phosphorylation and phosphorylation of large-conductance calcium-activated potassium (BKCa) channels (Figure 5). This results in the subsequent efflux of K+ ions through BKCa channels. This decreases cell contractility and volume and remodels the actin cytoskeleton of the TM and SC cells [59,60].

### 3.4. Rho Kinase Inhibitors

Rho kinases play pivotal roles in the modulation of AH outflow facility through the TM due to their critical inherent downstream effector activity on Rho GTPase signaling pathway. The Rho GTPase-mediated signaling pathway is recognized to control the formation of actin stress fibers, cellular contraction and relaxation, cytoskeletal organization, and focal adhesions [61,62]. Rho kinases (ROCKs) are a subgroup of serine/threonine specific protein kinases present in the TM and exist in two isoforms, namely ROCK1 and ROCK2. Inhibitors of Rho kinases such as ripasudil and netarsudil trigger actin–myosin interactions, to reduce extracellular matrix protein production, and disrupt both actin stress fibers and focal adhesions. This ultimately results in the relaxation of stiffened TM beams, thus primarily improving AH outflow. David Epstein and colleagues are among the leading researchers who led the discovery of cytochalasin and latrunculin, which were found to inhibit actin polymerization and cause disassembly of actin fibers [61]. Their findings propelled research into other Rho kinase inhibitors, including the synthetic ROCK inhibitor (Y-276332), which was shown to improve endothelial permeability, widen the circumference of Schlemm’s canal (SC) disrupt tight junctions, and F-actin depolymerization in SC cells [63]. Ripasudil (0.4%) is an example of a Rho kinase inhibitor that was approved for open angle glaucoma (OAG) and ocular hypertension in Japan. This was widely accepted and demonstrated moderate efficacy, resulting in 3–7 mmg IOP reduction with a peak onset within 12 h [64]. However, blepharitis and conjunctival hyperemia remained a reported side effect despite its transient nature. Netasurdil, on the other hand, was more recently approved by the FDA for OAG but offers an added advantage of combining the effect of both the ROCK inhibition along with norepinephrine transporter inhibition to target reduction in episcleral venous pressure and AH production [65].

### 3.5. Antioxidant Therapy

Given the apparent importance of oxidative stress in the pathogenesis of several disease processes, researchers have conducted considerable investigations in the development of effective antioxidants and potent RONS scavengers as possible therapeutic targets [12]. Antioxidants are compounds or naturally produced substances that inhibit oxidation by counteracting or slowing down the damage of cells against free radicals (oxidants). Antioxidants found in nature can be classified as enzymatic and non-enzymatic. Enzymatic antioxidants such as superoxide dismutase (SOD), glutathione reductase, glutathione peroxidase, and catalases are biological macromolecules that convert H_2_O_2_ to water in a multi-step process involving the use of cofactors such as iron, zinc, copper, and manganese. Naturally occurring non-enzymatic antioxidants produced in the body such as vitamin C, vitamin E, flavonoids, melatonin, curcumin, glutathione, flavonoids, and polyphenolic compounds possess intrinsic antioxidant properties [12,66].

### 3.6. Natural Antioxidants

Nutraceuticals are purported to possess medicinal properties including anticancer, antibacterial, antioxidants, wound healing properties, and reversing the course of neurodegenerative diseases [67]. Nutraceuticals belong to a remarkably diverse class of compounds normally found in everyday food constituents with potential benefits beyond their nutritional value. Natural antioxidants are often derived from plant sources and includes foods and spices such as green leafy vegetables (spinach), turmeric, rosemary (Rosmarinus officinalis), nutmeg (Myristica fragrans), epigallocatechin 3-gallate (EGCG) from green tea, resveratrol from grapes, and several others. These compounds possess active phytochemical components. Examples include carotenoids, vitamins A and E, phenolic acids (such as caffeic and cinnamic acid), curcuminoids, and flavonoids (genistein, quercetin). In vivo and in vitro studies involving nutraceuticals have demonstrated enormous potential as therapies for neurodegenerative diseases. In an in-vitro study conducted by Brunetti et al., allicin—an organosulphur responsible for the strong flavor of garlic (*Allium sativum* L.)—was examined on brain synaptosomes of old versus young rats. Their findings revealed that garlic extracts significantly decreased the production of 8-iso-prostaglandin F2α (8-iso-PGF) in young rats under hydrogen peroxide (H_2_O_2_)-induced oxidative stress when compared to control. Similarly, aged rat brain extracts showed a marked reduction in 8-iso-PGF among the H_2_O_2_-induced group [68]. In vivo studies have also shown remarkable roles of quercetin in mitigating cognitive impairments, ischemia, and traumatic injury. In addition, supplementation of quercetin (50 mg/kg) exerted antiseizure effects and enhanced memory in the cortex and hippocampus of pentyl tetrazole (PTZ)-kindled rats, however, generated ROS [69]. On the contrary, Carmona-Aparicio et al. suggested that quercetin demonstrated antiseizure and antioxidant effects and restored functions of antioxidant enzymes in kainic acid (induce temporal lobe epilepsy)-injected rats [70]. These contrasting results suggest that quercetin has both pro-oxidative and antioxidative effects; however, the detailed mechanisms in this regard are not clear. While natural antioxidants are considered a game changer in several diseases, there has been extensive work into structural modifications and isolation of chemically active antioxidant compounds with high ROS scavengers efficacy using multiple ways of targeting ROS damage.

### 3.7. Synthetic Antioxidants

**Figure 6:**
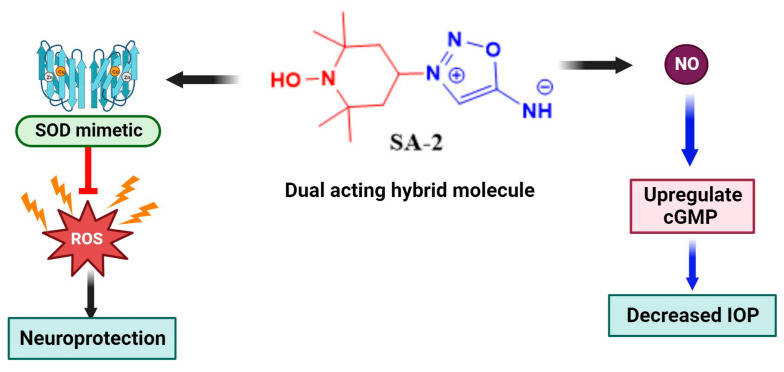
Proposed mechanism of action of synthetic hybrid NO donor and antioxidant small molecule SA-2 in ocular neurodegenerative disorders. The redox nitroxide antioxidant moiety 4-hydroxy TEMPOL (in red) acts as a superoxide dismutase (SOD) mimetic and inhibits oxidative damage in the trabecular meshwork, retina, and other ocular tissue. In particular, it scavenges superoxide radical and prevents the generation of peroxynitrite and depletion of NO bioavailability. The release of NO at physiologic concentrations from the sydnonimine NO prodrug (in blue) activates soluble guanylate cyclase to cause a downstream upregulation of cyclic GMP. This results in the relaxation of the trabecular meshwork, thus leading to a decrease in intraocular pressure and exerting neuroprotective effects.

Synthetic antioxidants such as 4-hydroxy TEMPO commonly known as TEMPOL is a stable cell membrane permeable cyclic nitroxide (molecular weight: 172.248 g/mol) previously utilized as a contrast material in magnetic resonance spectroscopy. In biological systems, TEMPOL exhibits properties like those of a potent metal-independent superoxide dismutase (SOD) enzyme. It has been shown to demonstrate neuroprotective effects, ROS scavenging activity, anticancer properties, and SOD mimetic activity in several pathological rodent models [71,72,73]. Evidence from Chiarotto et al. showed increased spinal cord motoneuronal survival (15–21%) and differential increase in caspase 3 (3-fold), Bax (13-fold), and Bcl2 (28-fold) gene expression after 12 h following a neonatal rat sciatic nerve transection model after TEMPOL treatment [74]. Similarly, TEMPOL was found to significantly reduce infarct volumes at doses of 20 mg/kg and 10 mg/kg, respectively, when compared with their controls (49.01 ± 18.22% reduction, *p* = 0.003 and (47.47 ± 34.57% reduction *p* = 0.02) during reperfusion in a rat model of transient focal ischemia [75]. In vitro human blood plasma and platelet samples obtained from young healthy non-smoking donors showed a significant reduction in peroxynitrite-mediated carbonyl group formation and thiobarbituric acid reactive substance (TBARS) formation post-treatment with TEMPOL, corroborating the antioxidant effect of TEMPOL (50 µM, 75 µM, and 100 µM) [76]. Other TEMPOL derivatives such as the water-soluble N-hydroxy-2,2,6,6-tetramethylpiperidine (**OT-440**) were shown to protect against loss of Thy-1 promoter activation following optic nerve crush after oral administration. Thy-1 is a cell surface protein expressed during the differentiation of RGCs. A direct comparison of Thy-1 positive retinal neurons showed that there were up to 60% more fluorescent retinal neurons in the mice that received **OT-440** than in corresponding control mice [77]. In the context of translational feasibility, TEMPOL-derivative OT-551 formulated as an eye drop was evaluated in phase II clinical trials for the management of geographic atrophy. **OT-551** maintained visual acuity and was well tolerated with few adverse events [78]. Despite the multifunctional properties of TEMPOL, it does not address the bioavailability of vasoactive NO. In principle, the structure of TEMPOL promotes ROS scavenging activity with no significant impact on vascular NO [79]. Furthermore, ROS generated from pathological states may cause a decrease in the amount of NO and thus induce dysfunction of the endothelium [79]. Recently, Acharya et al. designed and synthesized an innovative combination of TEMPOL and NO donating sydnonimine prodrug into one small molecule. The goal of this molecule is to reduce IOP in the trabecular meshwork and also provide additive antioxidant activity in the RGCs and ocular milieu under glaucomatous conditions [80,81]. This emerging hybrid NO donor and antioxidant TEMPOL derivatives (**SA-2**, **SA-9**, and **SA-10**) have been shown to exert cyto/neuroprotection. The proposed neuroprotection mechanism is mediated by multiple converging mechanisms including both direct and indirect detoxification of ROS in-situ and improving mitochondrial function. Hybrid molecule **SA-2** is shown to release NO at physiologic levels to upregulate c-GMP levels, which will in effect cause relaxation of the TM essentially lowering IOP. The tempol moiety on the other hand scavenges superoxide and a wide range of ROS (OH^.−^, HOCl, ONOO^.−^) to decrease progression of ROS-mediated apoptosis of the TM and retina (Figure 6) [81,82].

Unlike organic nitrates, which are poor nitric oxide donors in ocular tissues, 3-(4-morpholino)-sydnonimine hydrochloride (linsidomine, **SIN-1**), a mesoionic class of heterocyclic (1,2,3-oxadiazole amine) and a known NO donor is reported to effectively lower IOP in rabbit eyes [80]. However, **SIN-1** has a relatively short duration of action (3 h post dose) suggesting a limitation for in vivo efficacy. Stoichiometrically, **SIN-1** generates 1 mole of superoxide, which can react with NO to form peroxynitrite that can further trigger lipid peroxidation in ocular tissues.

Hybrid TEMPOL antioxidant and NO donating compound **SA-2** provide an added advantage of improving the bioavailability of NO in ocular tissues. This is achieved by scavenging superoxides, thereby preventing the generation of peroxynitrite and consequently preserving the bioavailability of NO. In biological systems, **SA-2** lowered IOP and demonstrated neuroprotection in both ex vivo hypoxic insult of adult rat retinal explants at doses (0.1, 0.5 and 1 mM) and improved RGC survival in the in vivo mouse optic nerve crush model at 1% dose after intravitreal injection [83,84].

In addition to providing neuroprotection, the hybrid compounds demonstrated significant cytoprotection to anterior segment of the eyes, including TM and increase outflow facility ultimately lowering IOP in rodent eyes with ocular hypertension (OHT). Isolated primary trabecular meshwork cells from both glaucomatous and healthy human donor eyes, showed a significant improvement in cell viability, improved mitochondrial metabolic parameters including oxygen consumption rate (OCR), and extracellular acidification rate (ECAR) with significant increase in antioxidant enzyme activities after **SA-2** treatment [22].

**Figure 7:**
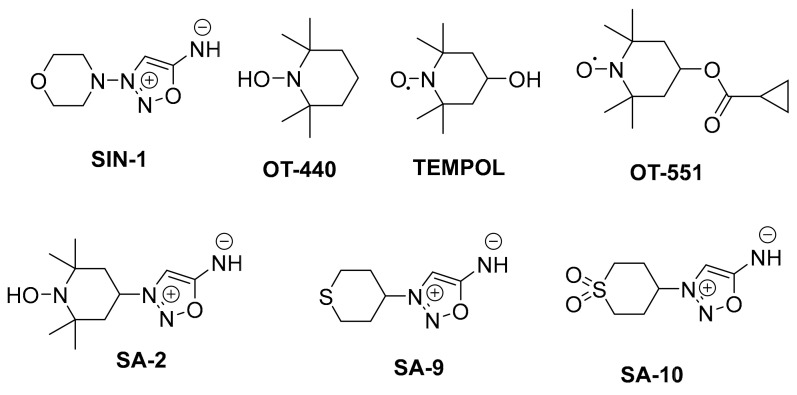
Chemical structures of SIN-1 (a known NO donor), OT-440, TEMPOL, OT-551 (a prodrug of TEMPOL), synthetic hybrid small molecules **SA-2, SA-9,** and **SA-10** containing NO donor and TEMPOL functionalities.

Similarly, the second-generation sulfide analog **SA-9** and its active oxidative sulfoxide metabolite **SA-10** showed improved antioxidant activity and potency in cytoprotection in TM cells than the first-generation hybrid compound **SA-2**. Both **SA-9** and **SA-10** are sulphur-containing compounds (Figure 7). Sulphur is known to occur in nature in some important amino acids and peptides including homocysteine, methionine, glutathione, and taurine. They participate in redox reactions, scavenging hydroxyl (OH^·^), superoxide (O_2_^·−^), as well as hypochlorous radicals (HOCl). Compounds **SA-9** and **SA-10** therefore offer more robust cellular protection, vasorelaxation, and longer duration of anticipated bioactivities [82].

Interestingly, just as **SA-2**, compounds **SA-9** and **SA-10** scavenge superoxide radicals reducing the formation of toxic peroxynitrites, thus maintaining NO bioavailability at physiologic levels sufficient to provide IOP-lowering activity in mouse model of OHT [82]. In addition, 1% of **SA-2** and 2% **SA-9** encapsulated in poly lactic co-glycolic acid (PLGA) nanoparticle (NP) formulation was reported to exert sustained IOP-lowering effect with considerable bioavailability in the choroid, sclera, retina, and lens [82,84]. In a separate experiment, **SA-10NP** restored blood perfusion in a peripheral arterial disease (PAD) model of chronic ischemia using Balb/c mice [85]. As expected, **SA-2** was also neuroprotective against endothelin-3, tertbutyl hydroperoxide (TBHP)-mediated insults and in trophic factor deprivation models of rat as well as human RGCs by reducing apoptosis and decreasing ROS levels (Table 1) [86].

## 4. Conclusions

In summary, the preclinical utilization of antioxidants and hybrid small molecules has gained prominence as viable therapeutic options for the management of ocular neurodegenerative diseases. The progression of these diseases such as glaucoma, AMD, and diabetic retinopathy are among the common causes of visual impairment globally. Given their multifactorial etiology, the discovery of multifunctional small molecules with antioxidant capability is vital for preserving visual function. With oxidative stress emerging as a significant player in the progression of neurodegenerative diseases, TEMPOL derivatives such as **SA-2**, specifically provides added advantage of enhancing bioavailability of NO, protecting against cellular apoptosis, inhibiting inflammatory responses, and accelerating neuronal repair mechanisms in many neurodegenerative diseases. However, further rigorous preclinical study in large animal eyes and human clinical trial studies will be essential in substantiating the reported benefits to advance safety and develop effective drug delivery modalities for ocular neurodegenerative diseases. Overall, antioxidants and hybrid TEMPOL derivatives such as **SA-2**, **SA-9**, and **SA-10** analogues offer promising therapeutic potential for improved visual outcomes either alone or in tandem with existing therapies as a comprehensive treatment strategy.

## Figures and Tables

**Table 1:** Summary table showing the overexpression or suppression of genes, proteins, or enzymes before and after treatment with TEMPOL/synthetic hybrid TEMPOL derivatives in several neurodegeneration models. Arrows pointing up indicate increase whereas arrows pointing down indicates decrease in respective proteins and/or enzymes.

Proteins/Enzymes	Changes Occur during Neurodegeneration	Changes after Treatment with TEMPOL or Hybrid TEMPOL Derivatives	References
Total antioxidants, Superoxide Dismutase, Glutathione peroxidase, Catalase,	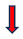	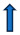	[22,72]
IL-1β, TNFα	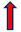	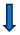	[87]
Malondialdehyde, Lipid peroxidation, TBARS	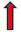	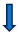	[22,76,82]
RGC count (injury models), TM viability Bcl-2	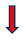	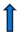	[74,75,84,86,88,89]
Caspase 3, 9, Bax, Bax/Bcl-xL ratio	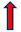	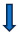	[74,89,90]

## Data Availability

Not applicable.
